# Supplementary material for: Influence of CYP2D6, CYP3A, and ABCG2 Genetic Polymorphisms on Ibrutinib Disposition in Chinese Healthy Subjects
Source: Pharmaceuticals (Basel). 2025 Oct 26;18(11):1615. doi: 10.3390/ph18111615 (PMC12655608; doi:10.3390/ph18111615)
Supplement: Supplementary file 1 [file pharmaceuticals-18-01615-s001.zip › pharmaceuticals-3830236-supplementary.pdf]

Figure S1 Representative LC–MS/MS chromatograms of sample types. (a) Blank plasma matrix; (b) plasma sample at the lower limit of quantification (LLOQ) with a ibrutinib concentration of 0.5 ng/mL; (c) plasma sample collected 1.67 hours after administration from fed subject C001. The blue trace shows the ibrutinib MRM transition (m/z 441.3→138.1), and the red trace corresponds to the internal standard (ibrutinib-d_5, m/z 446.3→309.1). Labeled peaks indicate their respective retention times.

a

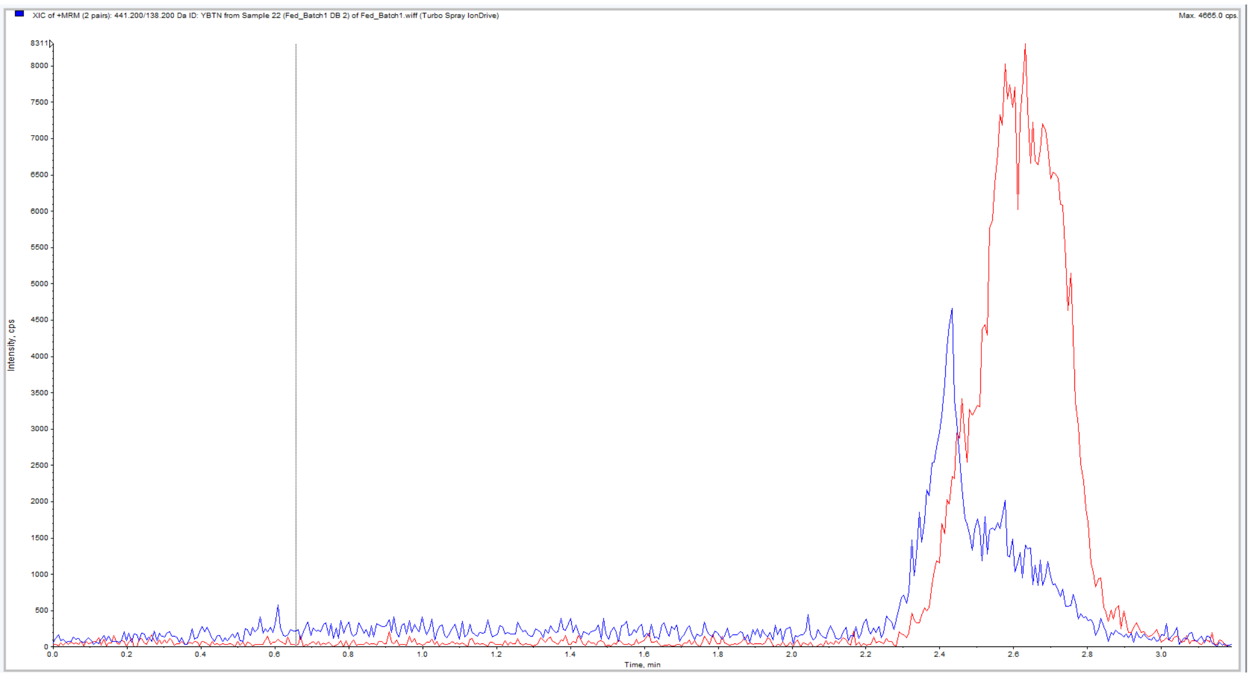

b

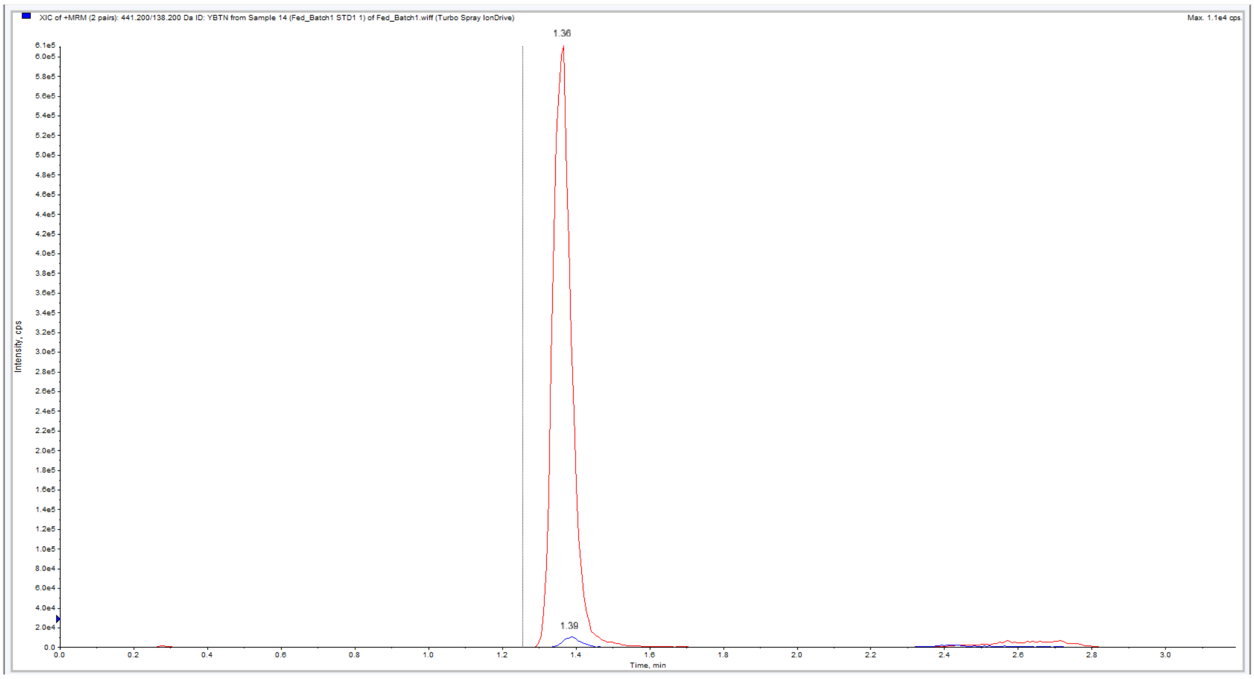

c

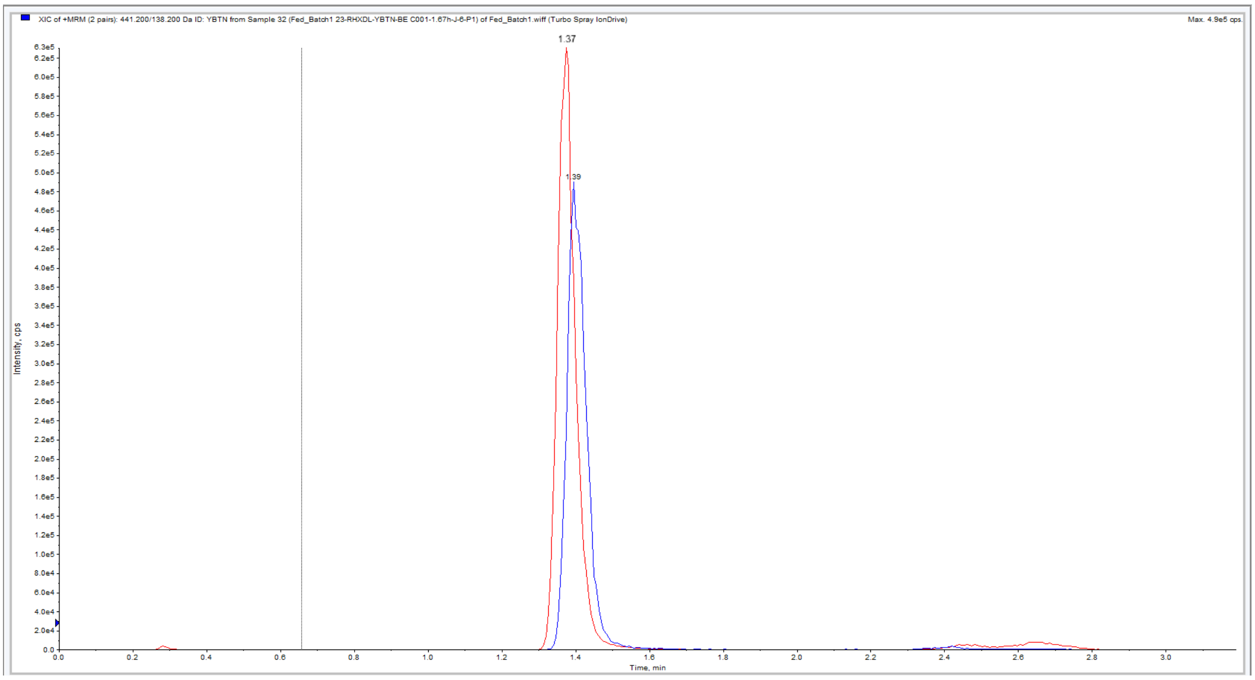

Table S1 Additional pharmacokinetic parameters of ibrutinib in healthy Chinese subjects separated by fasting and fed conditions.

Parameter	Fasting (N=16)	Fed (N=16)	P [1]
AUC0–∞ (ng·h·mL^{-1})	82.78 (53.65, 106.63)	114.26 (88.56, 153.38)	0.004**
λz (h^{-1})	0.09 (0.06, 0.23)	0.31 (0.23, 0.38)	0.000***
$t_{1/2}$ (h)	7.70 (2.95, 12.49)	2.27 (1.82, 3.04)	0.000***
Vz/F (L)	18727 (11060, 33827)	3984 (3204, 6610)	0.000***
% extrapolated AUC	11.20 (7.75, 22.43)	3.54 (2.81, 5.53)	0.000***

[1] Data were presented as median (the interquartile range). *P＜0.05, **P＜0.01, ***P＜0.001.
